# Remarks on Digestion

**Published:** 1851-11

**Authors:** N. P. Lattimore


### Part 3.—Selections.

ARTICEL I.

*Remarks on Digestion, suggested by the case of Carcinoma of the Stomach, reported in the August No. of this Journal.* By N. P. LATTIMORE, M. D.

In reading the interesting Report of Dr. P. H. Strong on "Carcinoma of the Stomach," published in the August number of your valuable Journal, I was struck with the first quere with which the doctor closes his communication. He says, "where was digestion carried on? Was it by the mucous coat, &c., or did the duodenum assume the function?

Taking the experiments of Mr. Bernard as a basis, the duodenum did not "*assume*" the function, for, according to him, digestion is *always* performed in the duodenum.

This doctrine is certainly not in accordance with the generally received notions upon this important question of physiology, yet, it is in direct accordance with experiment, and with observation, the two great bases upon which every truth must rest. M. Bernard, the pupil of the distinguished Magendie, although yet a young man, has enriched philosophy with many facts which do much to clear up some of the hitherto obscure points of this department of medical science. Made a member of the "Legion d' Honneur" for his discovery of the formation of sugar in the liver,—receiving the sanction of Magendie, Berard, and other eminent physiologists, his doctrines have become a part and parcel of our science; they only remain unknown on this side the Atlantic, because their author has not yet published them.

Where is digestion formed? Before answering this question, let us first determine what principles are the subject of this process. These may be reduced to three, viz: fibrine, sugar, and fat. These three matters are digested by means of certain secretions, viz., the saliva, the gastric juice, the bile, the pancreatic juice, and the intestinal secretion.

The office of the saliva seems to be to moisten the food during its mastication and swallowing, for we find it secreted in a direct ratio with the dryness of the masticated food. But upon this point we need spend no time.

After deglutition, the food enters the stomach, and is there subjected to the action of the gastric juice, which, like the saliva, is not secreted during abstinence. The gastric juice consists of

| | | |
|---|---|---|
| Water, | (parts) | 98 |
| Uncombined acid, | " | |
| Mucus, | " | |
| Phosphate of lime, | " | 2 |
| Pepsin, | " | |
| | | 100 |

The free acid is the lactic, and this, with the pepsin, are the active principles of the secretion.

Pepsin is an azotized substance, soluble in warm water, and if a few drops of acid are added to the solution, it will present all the properties of the gastric juice. This substance acts best at a temperature of 140°—that of the body during digestion.

Of the three alimentary principles, (fibrin, sugar, and fat,) the first, or fibrin, is the only one acted upon by the gastric juice, the other two passing into the duodenum unchanged.

In what manner does the gastric juice act upon the fibrin? Place a piece of meat in this secretion, and the first change observable is an increase of size, accompanied by a transparency of the edges. This change takes place as readily in acidulated water, as in the gastric juice, and is merely the physical effect of imbibition. But after a certain time (depending on the size of the ingested matters and the agitation to which they are exposed) the fibrin softens, and finally is wholly dissolved. Such is the action of the gastric juice upon all azotized ingesta; the non-azotized alimentary matters, as before remarked, are not affected by the secretion.

The result of this action is a fluid called chyme, and for a long time it was supposed that, in the production of this fluid, some chemical action between the gastric juice and the food was necessary. This opinion is now known to be erroneous, for if a portion of chyme resulting from the action of the gastric juice upon fibrin, be placed under the microscope, the existence of perfectly formed muscular fibres is evident; showing that the meat has been only disintegrated and not dissolved. No chemical change has as yet taken place, and the chyme is as really unfit for assimilation as was the fibrin itself.

After leaving the stomach, the chyme enters the duodenum, when it is subjected to the action of the bile, pancreatic juice, and intestinal secretion, and here it is that the most important part of the digestive process is accomplished. In man, the bile and pancreatic juice enters the duodenum by a common duct, and are thus mingled before their arrival in the small intestines. This is not true, however, in some of the lower animals, the rabbit, for example. In every case where these secretions are conveyed to the duodenum by separate ducts, the bile is first discharged. Thus in the rabbit, the hepatic duct enters the duodenum just below the pyloric orifice of the stomach, while the pancreatic duct comes in fifteen inches lower down. The most natural course, then, seems to be to consider, in the first place, the action of the bile upon the chyme, which it will be remembered consists of disintegrated fibrin, of fat, and of sugar; it is simply a solution of the ingesta in the gastric juice, and not a fluid *sui generis.*

Now the moment the chyme comes in contact with the bile, a precipitate is formed upon the intestinal villi, consisting of gelatin, albumen and casein, which have been dissolved in the gastric juice, united with the chloric acid of the bile. Then, *and not till then*, will these matters resist decomposition. Expose a portion of chyme to the action of the atmosphere, and in a few hours putrefaction will take place. If, however, a certain quantity of bile be mixed with the chyme, putrefaction is no longer possible. If yeast and sugar be mixed, fermentation immediately ensues, but it ceases the moment bile be added. The great office of the bile seems to be to prevent the decomposition of the alimentary matters which would necessarily take place without its presence. The chemical action which takes place during these changes, is not yet known. A simple experiment will show the influence of the bile in preventing fermentation; mix emulsina and amygdaline (both principles of the bitter almonds) and a decomposition results which produces prussic acid. If the two principles be thrown into the stomach together, the change takes place, and, if in sufficiently large quantities, death results. The same occurs if the amygdaline be administered by the mouth and the emulsine by the rectum; but reverse the process, give the emulsine by the mouth and the amygdaline by the rectum, and no prussic acid will be formed, because the bile coming in contact with the emulsine, removes its power of fermentation. Such, then, is the office of the bile.

*The pancreatic juice*, like the saliva and the gastric juice, is secreted only during digestion. In its action it cannot well be considered separately from the bile, because it always acts in conjunction with it, unless removed from the body. However, when so removed, it is found to dissolve fatty bodies as well as the azotized matters which have been precipitated by the bile, and also to convert the starch group of alimentary matters into sugar. Fat, during the digestive process, undergoes no chemical change, for we find it in the thoracic duct possessed of all the properties of fat; fat and lymph being the only constituents of chyle. How is fat capable of absorption? The gastric juice does not act upon it, nor does the bile, excepting to prevent its putrefaction. The only change it undergoes is a lessening of its globules, which are too large to be admitted into the lacteals. This diminuition of its globules is accomplished by the pancreatic juice. Obstruct in any way the pancreatic duct, and the fat, incapable of absorption, is carried off with the excrement, producing that form of diarrhea known as "adipose"—which is always present in cancer of the pancreas. Such, then, is the office of the pancreatic juice.

*En resume*, then, we see that the stomach only serves to prepare the alimentary matters for digestion; it does not act at all on fatty matters; it only soaks, or swells bodies of a starch group; its greatest action is on albuminous matters, and these it only partially dissolves, for in the solution of meat in the gastric juice the microscope still shows us muscular fibres.

The bile precipitates the solution just mentioned, and renders the whole incapable of putrefaction.

The pancreatic juice dissolves the precipitates formed by the bile; emulsions the fatty bodies; and changes bodies of the starch group into sugar.

But little is known of the action of the intestinal secretion, or of the secretion of the glands of Brunner. They are contained in the mucus membrane of the duodenum, and are of a more complex structure than any other glands in the vicinity. Brunner regarded them as accessory to the pancreas. Their influence upon digestion, however, is not fully known,

According to M. Bernard, then, the stomach acts upon alimentary matters only so as to prepare them for digestion, and it acts upon all bodies very nearly in the same way; while, for him, the duodenum is the grand center of the digestive process.

The case reported by Dr. Strong, comes to the support of this theory in more ways than one. Here digestion evidently could not have been performed in the stomach, and if that process was ordinarily accomplished here, the function would have been impaired, and thus attention long before have been directed to this organ.

Again, the limited capacity of the stomach satisfactorily accounts for the patient's craving only "the most concentrated diet."

The slight derangement of digestion might be premised when it was once known that neither the pylorus, nor the duodenum, nor the pancreas were involved in the disease;—the autopsy showing that the cancerous degeneration was almost wholly confined to the body of the stomach, none of the remaining viscera being involved excepting the colon, and that only slightly.

Trusting that I have satisfactorily replied to the quere, allow me to say, in conclusion, one word upon M. Bernard's views as to the ultimate disposition of the alimentary matters, or rather as to absorption. According to him, one only of the three principles, viz., fat, is taken up by the lacteals, and this it is which gives to chyle its white color. During abstinence the lacteals and the thoracic duct contain only lymph.

The other two principles, viz., fibrin and sugar, are absorbed by the veins, and their digestion completed in the liver. As they exist in the duodenum they are wholly unfit for assimilation, and it is only after passing through the liver that they are fitted for use by the economy.

M. Bernard's discoveries regarding the functions of the liver, perhaps constitute the most important contributions which have been made to physiology for the last half century, and an exposition of his views on this subject might interest many of your readers.—*Buffalo Med. Jour.*

